# Youth traffic-related injuries: a prospective study

**DOI:** 10.1186/s13017-016-0113-2

**Published:** 2017-01-05

**Authors:** Michal Grivna, Hani O. Eid, Fikri M. Abu-Zidan

**Affiliations:** 1 Institute of Public Health, College of Medicine and Health Sciences, UAE University, Al-Ain, United Arab Emirates; 2 Department of Surgery, Trauma Group, College of Medicine and Health Sciences, UAE University, Al-Ain, United Arab Emirates; 3 Department of Surgery, College of Medicine and Health Sciences, UAE University, Al-Ain, United Arab Emirates

**Keywords:** Youth, RTC, Traffic injury, Traffic safety

## Abstract

**Background:**

Traffic-related injuries are the most common cause of morbidity and mortality of the youth. Our aim was to study epidemiology, risk factors and outcome of hospitalized youth patients injured in road traffic collisions in order to give recommendations for prevention.

**Methods:**

We prospectively studied all youth (15–24 years) patients having traffic-related injuries who were admitted to Al Ain or Tawam Hospitals, Al Ain City, or who died after arrival to these hospitals during an 18 months period. Demography, location and time of injury, injured body regions, severity, hospital and intensive care unit (ICU) stay and outcome were analyzed.

**Results:**

Three hundred thirty-three patients having a mean age (SD) of 20 years (2.5) were studied. 87% were males and 72% were UAE nationals. Majority of injured patients were drivers or front-seat passengers (70%), followed by back seat passengers (16%), motorcyclists (5%) and pedestrians (4%). Rollover was the most common crash mechanism (35%), followed by front crash (34%). Twenty seven patients (8%) were ejected during the crash, 14 during roll-over, 7 from quadribikes and three during front crash. 20% of the patients were admitted to the ICU. Median Glasgow Coma Scale was 15 (range 3–15), median Injury Severity Score was 5 (range 1–41), and median total hospital stay was 3 days (range 1–73). Nine (3%) patients died.

**Conclusions:**

Young UAE-national males are at a higher risk of being injured at traffic. Rollover crash was frequent with high risk of ejection. Promotion of traffic safety and enforcement of safety legislation is necessary.

## Background

Traffic-related injuries are the most common cause of premature morbidity and a leading cause of death among the youth in the Middle-East [1, 2]. These injuries have a high impact on the affected victims, their families and societies [1]. According to the World Health Organization Global Status Report on Road Safety 2015 there are over 1.2 million road traffic deaths worldwide every year [3]. The estimated road traffic death rate in 2013 in the United Arab Emirates (UAE) was 10.9 per 100.000 population [3]. UAE is a fast developing country with a large proportion of young population. It has a growing number of vehicles (2.7 million in 2013) [3] and an expanding network of highways.

Specific risk factors for road traffic injuries in youth include inexperience, developmental changes with increased emotionality, overestimation of driving skills, increased risk taking, and response to peer pressure [4]. Prevention of road traffic collisions (RTCs), including use of safety belts and creating safe road environment, has been well-studied [5]. However risk factors vary in different settings. Despite legislation and increased enforcement in the UAE, the use of restraints among the youth is still very low [6]. Information on traffic-related injuries requiring hospitalization for this specific age group in our region is highly needed. We aimed to study the epidemiology, risk factors and outcomes of hospitalized road traffic injured youth patients in order to give recommendations for prevention.

## Methods

We prospectively studied all youth patients (15–24 year old) who were admitted to Al Ain City’s two major trauma centers or who died after arrival to these hospitals following RTCs during the period of April 2006 to October 2007. Al Ain City had about 460,000 inhabitants during study period [7]. Trauma patients were exclusively admitted to Al Ain Hospital and Tawam hospital. Al Ain hospital has 412 beds and provides a wide range of general and specialist clinical services [8], whereas Tawam Hospital is a highly specialized tertiary care center with 468 beds [9].

Patients or their caregivers were interviewed by a full time Research Fellow. We collected data on demography (age, gender, nationality), crash mechanism, place of injury, road user type, position in the vehicle, speed of the vehicle, use of safety equipment, time of the crash, anatomical body part(s) injured, severity, Revised Trauma Score (RTS), Glasgow coma scale (GCS), intensive care unit (ICU) admission, length of hospital stay, and outcome (survival or death).

Injury severity of different regions was calculated manually using The Abbreviated Injury Scale (AIS) of the Abbreviated Injury Scale Handbook [10]. This scale assigns each region a severity ranging from 1-6 (minor = 1, moderate = 2, serious = 3, severe = 4, critical = 5, unsurvivable = 6). The Injury Severity of the patients was assessed using the ISS [11]. The revised trauma score (RTS) was calculated using the systolic blood pressure, pulse rate, respiratory rate and GCS at arrival to the Hospital [12].

### Statistics

Nationality was divided into two categories (UAE nationals and non-UAE nationals) because the traffic risks differ between these two groups [6, 13, 14]. Comparison of continuous or ordinal data was performed using the Mann-Whitney U-test for two groups or the Kruskal-Wallis for more than two groups. Fisher’s exact test or Pearson Chi square test were used to compare categorical data of two or more independent groups as appropriate. A *p* value of less than 0.05 was needed to refuse the null hypothesis and accepting significant differences between the groups. Data were analyzed using Statistical Package for the Social Sciences (IBM-SPSS version 21.0, Chicago, Il, USA).

## Results

### Personal risk factors: gender, age and nationality

There were 333 patients, 290 males (87%). The mean age (SD) was 20 (2.5) years. Majority were UAE nationals (72%). The annual incidence of RTC hospitalizations using census data was estimated to be 279.4 per 100 000 person-years. Higher incidence was among males (411.1) than females (88.4). Although male to female population ratio was 1.5:1, the traffic-related injury ratio in our study was 6.7:1.

### Injuries by type of road user and vehicle type

Majority of injured patients were drivers or front-seat passengers (70%), followed by rear seat passengers (16%), motorcyclists (5%), and pedestrians (4%) (Table 1). The percentage of drivers were significantly higher among UAE nationals comapred with non-UAE nationals (*p* < 0001, Fisher’s Exact test). In contrast the percentage of rear seat passengers, pedestrians, and bicycle riders were significantly higher among non-UAE nationals comapred with UAE nationals (*p* = 0.04, *p* < 0.001, and *p* = 0.006 consequetivly, Fisher’s Exact test) (Table 1).

**Table 1:** Traffic-related youth injury hospitalisations by road user type and nationality, Al Ain, 2006–2007 (*n* = 333*)

Road user	UAE (*n* = 238)		Non-UAE (*n* = 94)		*p*-value	Total (*n* = 332)	
	Number	%	N	%		N	%
Vehicle Occupant							
Driver	129	54.2	26	27.7	*P* < 0.0001	155	46.7
Front seat	52	21.8	25	26.6	0.39	77	23.2
Rear seat	31	13	21	22.3	0.044	52	15.7
Vulnerable road user							
Pedestrian	2	0.8	13	13.8	*P* < 0.0001	15	4.5
Cyclist	0	0	4	4.3	0.006	4	1.2
Motorcyclist	13	5.5	4	4.3	0.79	17	5.1
Quadrubike	11	4.6	1	1.1	0.19	12	3.6

*p* Fisher’s Exact test

*Information on road user type was missing in 1 patient

Numbers may not add to 100 due to rounding

There was no significant difference in age, GCS, RTS and ISS between vehicle occupants and vulnerable road users (pedestrians, bicyclists, motorcyclists, and quadribike users). Mortality among vehicle occupants was 2% compared with 4% in vulnerable road users.

Motorcyclists and cyclists were all males (100%). Table 2 compares those patients who were less than 18 years old and those who were ≥18 years old. Back seat passengers and motorcyclists were significantly higher in those less than 18 years old (*p* < 0.001, Fisher’s Exact test). Twelve drivers (8%) and 6 motorcyclists (35%) were under the licensing age in the UAE (18 years old). There were also 6 (50%) quadrubike users less than 18 years old. Underaged motorcyclists were injured off-road and in the parking or housing areas. Two underaged quadrubike users were injured on highway or street while four were injured off-road.

**Table 2:** Demographic and severity variables by age category (<18 and ≥18), Al Ain, 2006–2007, *n* = 333

	Age <18 years (*n* = 62)	Age ≥18 years (*n* = 271)	*p*-value
Gender (male)	53 (85.5%)	237 (87.5%)	0.68
UAE nationals	49 (79%)	189 (69.7%)	0.16
Type of patient			*p* < 0.0001
Driver	12 (19.4%)	143 (53%)	
Front seat	18 (29%)	59 (21.8%)	
Back seat passenger	18 (29%)	34 (12.6%)	
Motorcyclist	6 (9.7%)	11 (4.6%)	
Quadrubike user	6 (9.7%)	6 (2.2%)	
Cyclist	0	4 (1.5%)	
Pedestrian	2 (3.2%)	13 (4.8%)	
ICU admission	12 (19.4%)	52 (19.2%)	0.99
Hospital stay (days)	15 (2–69)	3 (1–58)	0.25
Mortality	1 (1.6%)	8 (2.95%)	0.7
GCS	9 (5–15)	15 (3–15)	0.42
RTS	10 (8–12)	12 (7–12)	0.44
ISS	5 (1–41)	5 (1–41)	0.31

Data are presented as number (%) or median (range) as appropriate

*p* Fisher’s Exact test or Mann Whitney test as appropriate, *ICU* Intensive Care Unit, *GCS* Glasgow Coma Scale, *RTS* Revised Trauma Score, *ISS* Injury Severity Score

Fifty three percent (150/285) vehicle occupants were injured in sedan cars, 44% (124/285) in sport utility vehicles (SUVs) and 4% (11/285) in other vehicles. Male drivers were significantly more injured driving SUVs compared with females (70/148 (39%) comapred with 0/6 (0%), *p* = 0.032, Fisher’s Exact test). Females were driving only sedan cars. Sixty six percent (103/155) of drivers were driving alone; 64% (7/11) of drivers who were less then 18 years old were driving alone.

### Crash mechanism

Rollover of the vehicle was the most common crash mechanism of injury (35%), followed by front impact collision (34%) (Table 3). Secondary roll-over of the car was in 50% of rear-end, 37% of side and of 18% of front-impact crashes. Twenty seven patients (8%) were ejected during the crash. More UAE nationals were injured in rollover crashes compared with front or side angle (*p* = 0.002) (Table 3). Patients in rollover crashes had a longer stay in the hospital compared with front and side angle crashes (*p* = 0.03) (Table 3).

**Table 3:** Demographic and severity variables by car crash mechanism, Al Ain, 2006–2007 (*n* = 333)

	Car crash mechanism				
	Front	Side angle	Rollover	Other	*p*-value
	*n* = 96	*n* = 57	*n* = 98	*n* = 29	
Age (years)	20.2 (2.2)	20.7 (2.6)	19.8 (2.3)	18.9 (2.3)	0.006
Gender (male)	84 (87.5%)	45 (78.9%)	87 (88.8%)	27 (93.1%)	0.26
UAE national	69 (71.9%)	35 (61.4%)	85 (86.7%)	19 (65.5%)	0.002
ICU admission	19 (19.8%)	11 (19.3%)	21 (21.4%)	5 (17.2%)	0.28
Hospital stay (days)	2 (1–68)	3 (1–73)	5 (1–58)	2 (1–44)	0.03
Mortality	2 (2.1%)	2 (3.5%)	1 (1%)	1 (3.4%)	0.72
GCS	15 (5–15)	15 (4–15)	15 (3–15)	15 (7–15)	0.1
RTS	12 (9–12)	12 (9–12)	12 (7–12)	12 (10–12)	0.87
ISS	5 (1–45)	5 (1–34)	5 (1–36)	5 (1–41)	0.29

Data are presented as number (%), mean (SD) or median (range) as appropriate

Other mechanisms include back crash or crash with motorcycle or bicycle

*p* Fisher’s Exact test, Pearson chi square, or Kruskall Wallis test as appropriate, *ICU* Intensive Care Unit, *GCS* Glasgow Coma Scale, *RTS* Revised Trauma Score, *ISS* Injury Severity Score

### Speed of the car

**Fig. 1:**
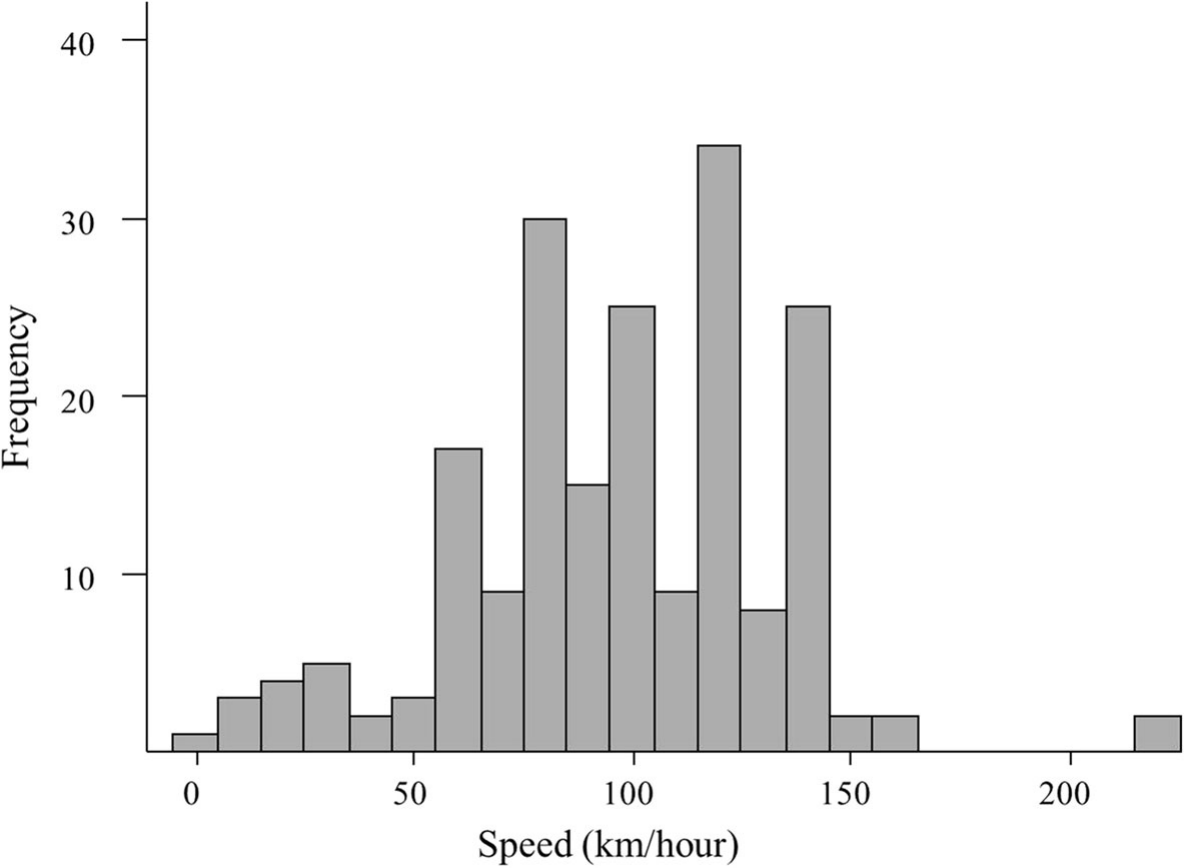
Distribution of speed of the cars involved in road traffic collision (*n* = 196)

The mean (SD) of car speed was 97.2 (35.8) km/hr, 42% were higher than the legal speed limit of 100 km/hr (Fig. 1).

### Place and time of injury

Majority of traffic-related injuries occurred on highways and streets (276/333;83%), 7% (24/333) off road, 6% (20/333) around homes in residential areas, and 4% (13/333) in other locations. Thirty three percent (5/15) of pedestrians and 29% (5/17) of motorcyclists were injured in housing areas. Seventy five percent of quadrubike users (9/12) and 24% of motorcyclists (4/17) were injured off road.

**Fig. 2:**
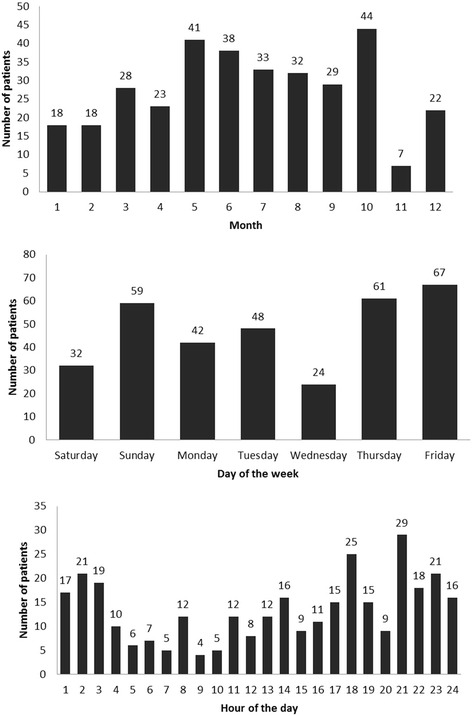
Distribution of patients by month, day and hour (*n* = 333)

Evening (6–12 pm) was the most common time of crashes (34%) and Friday the most common day of crashes (20%) (Fig. 2). Most of injuries occured in the period of May to October.

### Safety equipment, distraction, sleep and alcohol

Only 12% (*n* = 18) of the drivers, and 4% (3) of front seat passengers were restrained. No back seat passenger used a setabelt. Five motorcyclists used a helmet (17%) and two wore protective clothing (7%). No byclist or quadrubike user used a helmet. Eight drivers (5%) were using mobile phones. Seven drivers were sleepy when they crashed (4%). Alcohol use was found only in one patient.

### Severity and anatomical location of injuries

There were 66 patients (20%) admitted to the ICU. Median GCS was 15 (range 3–15), median ISS was 5 (range 1–41), median RTS was 12 (range 7–12) and median total hospital stay was 3 days (range 1–73). Nine patients (2.7%) died.

The head was the most common injured region (67%) followed by extremities and chest (Table 4). The highest AIS score was in the chest (mean AIS 2.5) followed by the spine (mean AIS 2.3) (Table 4). Eighty percent of ejected patients sustained a head injury.

**Table 4:** Traffic-related youth injury hospitalisations by anatomical region and AIS severity, Al Ain, 2006–2007 (*n* = 333)

Anatomical Region	Number (%)		Injury severity by Maximum AIS*			
			Mean	Median	Min	Max
Head	223	67.0	1.91	2	1	5
Chest	107	32.1	2.50	3	1	5
Abdomen	47	14.1	1.77	1.5	1	4
Spine	43	12.9	2.30	2	2	5
Upper extremity	114	34.2	1.64	2	1	3
Lower extremity	135	40.5	1.79	2	1	3
Superficial	44	13.2	1.02	1	1	2

*Maximum Abbreviated Injury Scale – only the most severe injury per body region was counted for each patient; Some patients have injury in more than one region

## Discussion

Youth is the active period of life with major developments affecting adult health [2]. Traffic-related injuries are the most common cause of morbidity and mortality in the youth. In our study, young UAE-national males were at higher risk of being injured in traffic. Rollover crash was common with a high risk of ejection. Restraint use was extremly low in our study population.

The youth male preponderance has been described in many studies [2, 3, 15, 16]. Young male drivers have a higher collision rate than women [5] and their death rate is double compared with women [17]. In the UAE, young women drive less and usually drive small cars, which are less prone to rollover. During the cognitive development the ability of youth to make safe decisions on the road is not mature [16, 18]. Adolescents are known to seek out risks when driving [4]. They have lower compliance with restraint use in our region [6, 16].

Despite legislation and increased law enforcement, seat belt use remains low in our setting, especially among young UAE nationals [6]. Only 12% of drivers and 4% of front seat passengers were restrained in our study. This poses a serious risk to all vehicle occupants. There is a high risk of severe injury and fatality for unrestrained passangers, especially in front collisions and rollovers [13].

Rollover crash was the most common crash mechanism in our study with 8% of the passengers being ejected. SUV is a very popular vehicle in the UAE, especially among UAE national families, who like to drive in the desert and off-road. These cars tend to roll-over during collision, because they have a higher center of gravity. Ejection rate is high because of the low use of restraints in our community. Quadrubike use by teenagers in our study caused 4% of all crashes, 75% of them sustained a head injury. Head injury was also common among other vulnerable road users (bicyclists and motorcyclists) who did not use helmets. Bicyclists in our study were only non-UAE nationals. They are usually poor workers using bicycles for their transport. The percentage of pedestrian injuries were also higher among non-UAE nationals comapred with UAE nationals. UAE nationals tend to use the car even for very short distances compared with non-UAE nationals who generally walk to perform their duties.

Distractive driving is a major contributing factor for traffic collisions. US Transportation Department reported that nearly 20% of all crashes involve some distractive driving [19]. Distractive driving causes impairement in driving performance and prolongs reaction time [20]. In our study, 5% of drivers were using mobile phones when they crashed. Despite legislation, use of mobile and smart phones while driving is common in the UAE. Alcohol use in the youth was less than 0.5% in the present study. Overall alcohol use in road traffic injuried patients in our city was 2.1% [21]. This is attributed to legislation, religious believes, and limited accessibility to alcohol.

The age for obtaining driving license in the UAE is 18 years. 8% of injured drivers in our study were younger than this age and 64% of them were driving alone when they crashed. Families ignore the need for a license for short trips and use adolescents for bringing siblings from school and for shopping. A study in Oman found that 33% of students had driven without a license and 34% liked to speed [22]. 35% of motorcycle riders and 50% of quadrubike riders were under 18 years old in our study. Unlicensed driving is a major problem for traffic safety as it often correlates with high-risk behaviors such as speeding, failure to wear a seat belt or motorcycle helmets. Unlicensed drivers were three times more likely to be involved in a collision than licensed drivers [23].

Most of our patients were injured at night (64%). This is possibly contributed to impaired visibility during night and a possibility to drive with high speed because of presence of fewer vehicles. Violation of speed limits is another important contributing factor to RTCs [5]. The traffic design in Al Ain with long roads having 3 lanes between roundabouts allows the youth to speed. Drivers who drive faster or slower than the mean speed of traffic have a higher risk of crash [5].

There was a sharp increase of injuries during the weekend (Thursday and Friday) and in October which was the fasting month of Ramadan during the study period. The Canadian study [24] which assessed the increased crashes during the weekends and holidays, found three main risky behaviors: unsafe speeding, non-use of restraints, and driver intoxication. There is increased risk for traffic collisions during the fasting month of Ramadan, epecially in the evenings when tired drivers after a day without food or drinks are rushing home for breaking fast [25, 26]. Sleepeness occured in 4% of drivers involved in road traffic collisions in our city. This risk increased during the fasting month of Ramadan [26].

The police, transportation and the health sectors in the UAE have made active efforts to reduce the burden of RTCs over last decades. This included introduction of new laws and regulations, enforcement of speed limits, improved road design, and educational campaigns [27]. Plans are underway to introduce mandatory seat belts for rear seat passengers and child safety restraints [27].

### Limitations of the study

We have to acknowledge that there are certain limitations in our study. We studied only patients who were admitted to the hospital or those who died in the Emergency Department following road traffic collisions. More seriously injured patients may have died before arriving to our hospitals. Furthermore, our study population was from Al Ain City, limiting the generalizability of our results for other parts of the UAE. Finally, our study was a specific time limited research project supported by the UAE University before 2007. It may be questioned whether our results reflect the present situation. We think that risk factors for youth traffic injuries are still the same in our city.

## Conclusions

Young UAE-national males are at a higher risk of being injured at traffic. Rollover crashes have high risk of ejection. There is a need for implementation of cultural relevant evidence-based educational programs for all new and existing youth drivers. Promotion of traffic safety and enforcement of safety legislation is neccesary. Distractive and underage driving should be controlled.

## References

[1] Al-Kharusi W (2008). Update on road traffic crashes: Progress in the Middle East. Clin Orthop Relat Res.

[2] Makhlouf Obermeyer C. Adolescents in Arab countries: Health statistics and social context. DIFI Family Research and Proceedings 2015:1. http://www.qscience.com/doi/pdf/10.5339/difi.2015.1. Accessed 4 Sep 2016.

[3] WHO. Global status report on road safety 2015. Geneva, World Health Organization; 2015. http://www.who.int/violence_injury_prevention/road_safety_status/2015/en/. Accessed 4 Sep 2016.

[4] Arnett J (2002). Developmental sources of crash risk in young drivers. Inj Prev.

[5] Elvik R, Hoye A, Vaa T, Sorensen M. The handbook of road safety measures. 2nd ed. Bingley, UK: Emerald Group Publishing Limited 64. 2009. p.172-173.

[6] Barss P, Al-Obthani M, Al-Hammadi A, Al-Shamsi H, El-Sadig M, Grivna M (2008). Prevalence and issues in non-use of safety belts and child restraints in a high-income developing country: Lessons for the future. Traffic Inj Prev.

[7] Ministry of Health UAE. Annual Report 2008; 2008:4.

[8] Al Ain Hospital. 2016. https://www.seha.ae/alain/English/Pages/default.aspx. Accessed 4 Sep 2016.

[9] Tawam Hospital. 2016. https://www.seha.ae/tawam/English/Pages/default.aspx. Accessed 4 Sep 2016.

[10] Association of the Advancement of Automotive Medicine. Abbreviated Injury Scale. Association for the Advancement of Automotive Medicine, Barrington, IL. 1998

[11] Bergeron E, Lavoie A, Moore L, Bamvita JM, Ratte S, Clas D (2006). Paying the price of excluding patients from a trauma registry. J Trauma.

[12] Maurer A, Morris JA Jr. Injury Severity Scoring. In: Moore E, Feliciano D, Mattox K (eds) Trauma (5th ed.), McGraw-Hill Companies, Inc, New York. 2004. p. 87-91.

[13] Abu-Zidan FM, Abbas AK, Hefny AF, Eid HO, Grivna M (2012). Effects of seat belt usage on injury pattern and outcome of vehicle occupants after road traffic collisions: prospective study. World J Surg.

[14] Hefny AF, Barss P, Eid HO, Abu-Zidan FM (2012). Motorcycle-related injuries in the United Arab Emirates. Accid Anal Prev.

[15] Mansouri FA, Al-Zalabani AH, Zalat MM (2015). Road safety and road traffic accidents in Saudi Arabia – A systematic review of existing evidence. Saudi Med J.

[16] Sarhan NA (2012). Non-intentional injuries in adolescents and youth: Facts and figures. Bahrain Med Bull.

[17] NHTSA (National Highway Traffic Safety Administration). Traffic Safety Facts 1997. Overview, U.S. Department of Transportation, Washington DC. 1997. Accessed on April 18, 2016 from www-nrd.nhtsa.dot.gov/Pubs/TSF1997.PDF. Accessed 4 Sep 2016.

[18] Dunbar G, Hill R, Lewis V (2001). Children’s attentional skills and road behaviour. Exp Psychol Appl.

[19] NHTSA (National Highway Traffic Safety Administration). Distracted driving 2009. In Traffic Safety Facts – Research Note. 2010. http://www-nrd.nhtsa.dot.gov/Pubs/811379.pdf. Accessed 4 Sep 2016.

[20] WHO. Mobile phone use: a growing problem of driver distraction. Geneva, Switzerland, World Health Organization. 2011. file:///C:/Users/m.grivna/Documents/1UAEU/2Publications/Journals/Youth%20injuries/References/2011%20WHO%20-%20NHTSA%20-%20Mobile%20phone%20use%20-%20A%20growing%20problem%20of%20driver%20distraction.pdf. Accessed 4 Sep 2016.

[21] Osman OT, Abbas AK, Eid HO, Salem MO, Abu-Zidan FM (2015). Alcohol-related road traffic injuries in Al-Ain City, United Arab Emirates. Traffic Inj Prev.

[22] Jaffer YA, Afifi M, Al Ajmi F, Alouhaishi K (2006). Knowledge, attitudes and practices of secondary-school pupils in Oman: I. health-compromising behaviours. East Mediterr Health J.

[23] Watson B, Steinhardt D. A comparison of the crash involvement of unlicensed motorcycle riders and unlicensed drivers in Queensland. In Proceedings 2006 Australasian Road Safety Research, Policing, Education Conference, Gold Coast, Quensland. 2006. http://eprints.qut.edu.au/5457/1/5457.pdf Accessed 14 Dec 2016.

[24] Anowar S, Yasmin S, Tay R (2013). Comparison of crashes during public holidays and regular weekends. Accid Anal Prev.

[25] Mehmood A, Khan IQ, Mir MU, Moin A, Jooma R (2015). Vulnerable road users are at greater risk during Ramadan – results from road traffic surveillance data. J Pak Med Assoc.

[26] Al-Houqani M, Eid HO, Abu-Zidan FM (2013). Sleep-related collisions in United Arab Emirates. Accid Anal Prev.

[27] Grivna M, Aw TC, El-Sadeg M, Loney T, Sharif A, Thomsen J, Mauzi M, Abu-Zidan FM (2012). The legal framework and initiatives for promoting safety in the United Arab Emirates. Int J Inj Control Saf Promot.

